# A new species from Thailand and Burma, *Dracaena kaweesakii* Wilkin & Suksathan (Asparagaceae subfamily Nolinoideae)

**DOI:** 10.3897/phytokeys.26.5335

**Published:** 2013-10-02

**Authors:** Paul Wilkin, Piyakaset Suksathan, Kaweesak Keeratikiat, Peter van Welzen, Justyna Wiland-Szymanska

**Affiliations:** 1 Royal Botanic Gardens, Kew, Richmond, Surrey, TW9 3AB, UK; 2 Queen Sirikit Botanic Garden, P.O. Box 7, Mae Rim, Chiang Mai 50180, Thailand; 3 264/45-46 Suksawad 15, Suksawad Rd., Rajaburana, Bangkok 10140, Thailand; 4 Naturalis Biodiversity Center, section NHN, Leiden University, P.O. Box 9514, 2300 RA Leiden, The Netherlands; 5 Department of Plant Taxonomy, A. Mickiewicz University, Ul. Umultowska 89, 61-614 Poznań, Poland

**Keywords:** *Dracaena* L., dragon trees, Thailand, Burma, taxonomy, morphology, conservation

## Abstract

A morphologically distinct element of the group of *Dracaena* species from Thailand and Burma with undifferentiated leaf sheaths, no leaf blade central costa, free tepals and free thickened filaments known as *Chan nuu* or *Chan pha krai* in Thai is shown to be a distinct species, *Dracaena kaweesakii* Wilkin & Suksathan based on habit, leaf base and margin, inflorescence axis indumentum and floral characters. It is described and illustrated. Ecological and conservation status assessment information are provided.

## Introduction

The taxonomy of the “dragon tree” group of *Dracaena* L. species in mainland South-East Asia was summarised in Wilkin et al. (2012). The species possess an undifferentiated leaf sheath, lack a leaf blade central costa and have free tepals and free thickened filaments. Three species with this set of characters are recognised in the World Checklist of Monocotyledons (WCM, Govaerts et al. 2011), *Dracaena cambodiana* Pierre ex Gagnep., *Dracaena cochinchinensis* (Lour.) S.C.Chen and *Dracaena yuccifolia* Ridl. The WCM suggests that there are a total of 15 species of *Dracaena* in Thailand. A further “dragon tree” species occurs in South-East Asian and Pacific islands, *Dracaena multiflora* Warb. ex Sarasin (Wong et al. 1999).

In addition to the three accepted names in Govaerts et al. (2011) and *Dracaena jayniana* Wilkin & Suksathan that was described in Wilkin et al. (2012), a further element of the “dragon tree” group of species usually known as *Chan nuu* or *Chan pa krai* in Thai appeared distinct in its macromorphology from all other related taxa. Thus it was investigated more closely to discover if it represented an undescribed taxon.

## Materials and methods

This research involved comparative morphological study of living plants of the “dragon tree” group of *Dracaena* in the field and in cultivation in Thailand and of the specimens cited below. Further specimens of *Dracaena* from the herbaria BM, BK, BKF, QGB, C, K, L, P and WAG were examined. In order to test the hypothesis that *Chan nuu/Chan pa krai* represented a distinct taxon it was compared directly with *Dracaena yuccifolia* (Ridley 1896) and with *Dracaena cambodiana*, *Dracaena cochinchinensis*, *Dracaena jayniana* and *Dracaena multiflora*. The principal characters used are listed in Table 1.

**Table 1.** A summary of the main morphological character state differences between *Dracaena kaweesakii* and *Dracaena yuccifolia* Ridl.

Character	*Dracaena kaweesakii*	*Dracaena yuccifolia*
Habit	To 12 m tall, branches to several hundred	To 6 m, usually not more than 3 m in coastal forms; branches to ca 80.
Leaf blade dimensions (mm)	110–605 × 9–31	90–735 × 7–35
Leaf blade texture	Thickly chartaceous to thinly coriaceous	Thinly coriaceous
Leaf base colour	White, when fresh, pale to mid brown when dry	Often yellow-hued or brown in living and dried material
Leaf margin	Dark to mid green with a ca 1-2 mm white margin when fresh	Pale to mid green, margin concolorous
Inflorescence axis indumentum	Tuberculate-villous, often dense, sometimes crisped, to 0.15 mm long	Absent to microaculeate, trichomes ca 0.01 mm long
Floral stalk dimensions (above articulation at pedicel apex)	Floral stalk absent (i.e. flower inserted directly on articulation at pedicel apex)	0.5–0.8 × 1.1–1.3 mm
Tepal colour	Cream with a green or yellow hue, paler and more translucent towards margins, greener towards apex and along costa	Bright white
Filament length (mm)	2.2–3.8	3.3–4.9
Filament colour	Intense orange	Bright white
Filament orientation	Erect	Spreading
Anther length (mm)	1.7–2.2	1.0–1.4
Style length (mm)	2.5–3.3	3.4–5.5
Ovary dimensions (mm)	2.3–3.3 × 1.3–2.0	1.8–3.2 × 0.5–1.1
Fruit colour at maturity	Brown, becoming orange just before or after falling from infructescence	Dull red on infructescence
Distribution	Thailand (Saraburi to Chiang Rai) and adjacent eastern Burma.	Thailand (Ratchaburi) to Malaysia (Langkawi)

## Results

Both *Dracaena yuccifolia* and *Chan nuu*/*Chan pa krai* differ from other taxa of the Asian “dragon tree” group of *Dracaena* species in their much-branched habit (there may be several hundred branches in large mature trees of *Chan nuu*), with spreading and dividing branches usually borne on a short, unbranched basal trunk. They also possess narrow terminal branches, usually less than 2 cm in diameter near the leafy apices. The other taxa, *Dracaena cochinchinensis*, *Dracaena cambodiana*, *Dracaena jayniana* and *Dracaena multiflora* (Gagnepain 1934, Wong et al. 1999, Chen and Turland 2000) usually have not more than 10 erect or ascending stems bearing few erect to decumbent branches which are not spreading. Their terminal branches are more robust, being at least 2.4 cm in diameter. The latter taxa also possess thicker leaf blades with stronger longitudinal costae than *Dracaena yuccifolia* and the entity under study, in which the leaf blades are relatively thin and soft.

Table 1 shows the main morphological differences between *Dracaena yuccifolia* and *Chan nuu/Chan pa krai*. It is clear that the habit, vegetative and reproductive morphology of the latter are distinct from that of the former species. Thus it is described as a new species below.

## Description

### Dracaena kaweesakii

Wilkin & Suksathan sp. nov.

urn:lsid:ipni.org:names:77132456-1

http://species-id.net/wiki/Dracaena_kaweesakii

Figs 1 [Fig F2] –3

#### Diagnosis.

*Dracaena kaweesakii* differs from *Dracaena yuccifolia* Ridl. in its habit, with up to several hundred branches, white (brown when dry) leaf sheaths lacking yellow or dark brown pigmentation and blades with a narrow white margin when fresh. The inflorescence axis of *Dracaena kaweesakii* is tuberculate-villous, and the species lacks a floral stalk above the pedicel articulation. The tepals are cream-green or cream-yellow and the filaments intense orange. The anthers are 1.7–2.2 mm long and the style 2.2–3.3 mm. The ovary is 1.3–2.0 mm broad and develops into and fruits that largely remain brown on the infructescence, turning orange only just before or after falling.

#### Type.

**Figure 1.**
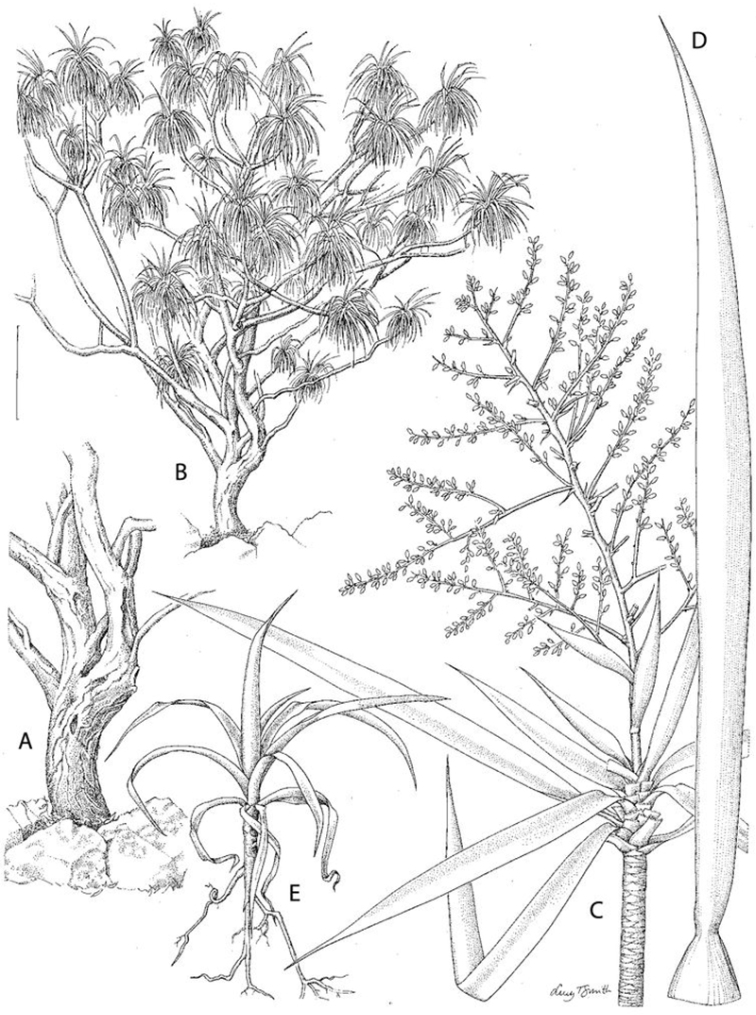
The habit, vegetative, inflorescence and seedling of *Dracaena kaweesakii*. **A** Trunk base showing the pattern of branching and corky, fissured surface **B** Habit **C** Branch apex with erect inflorescence **D** Leaf sheath and blade **E** Seedling with rosulate leaves. Scale bar: **A** 50 cm; **B** 125 cm; **C** 6 cm; **D** 3 cm; **E** 4 cm. From *Maxwell* 96-379 (**C**), *Geesink* et al. 8122 (**D**) and photographs. Drawn by Lucy Smith.

**Figure 2. F2:**
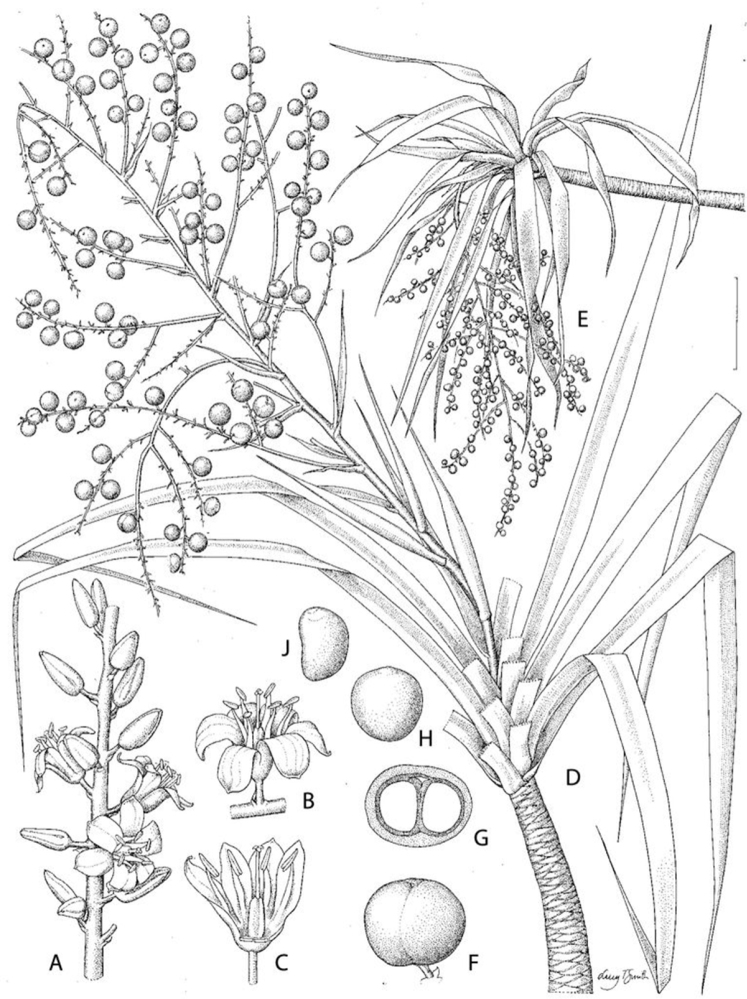
Reproductive organ morphology of *Dracaena kaweesakii*. **A** Part of partial inflorescence bearing solitary flowers **B** Flower in side view showing tepal shape and orientation and stamens **C** Flower with 2 tepals removed showing filaments shape and insertion and gynoecium **D** Branch apex showing infructescence in development and peduncular bracts **E** Branch apex with infructescence rendered pendent by weight of fruit **F** Submature fruit containing two seeds **G** The same fruit in cross-section **H, J** seed in dorsal and side view respectively. Scale bar: **A, F, G** 1 cm; **B, C, H, J** 7 mm; **D** 4 cm; **E** 10 cm. From *Siriponamat 2* (**A**), *Siriponamat* 3 (**D**), *Geesink* et al. 8122 (**F–J**) and photographs. Drawn by Lucy Smith.

**Figure 3.**
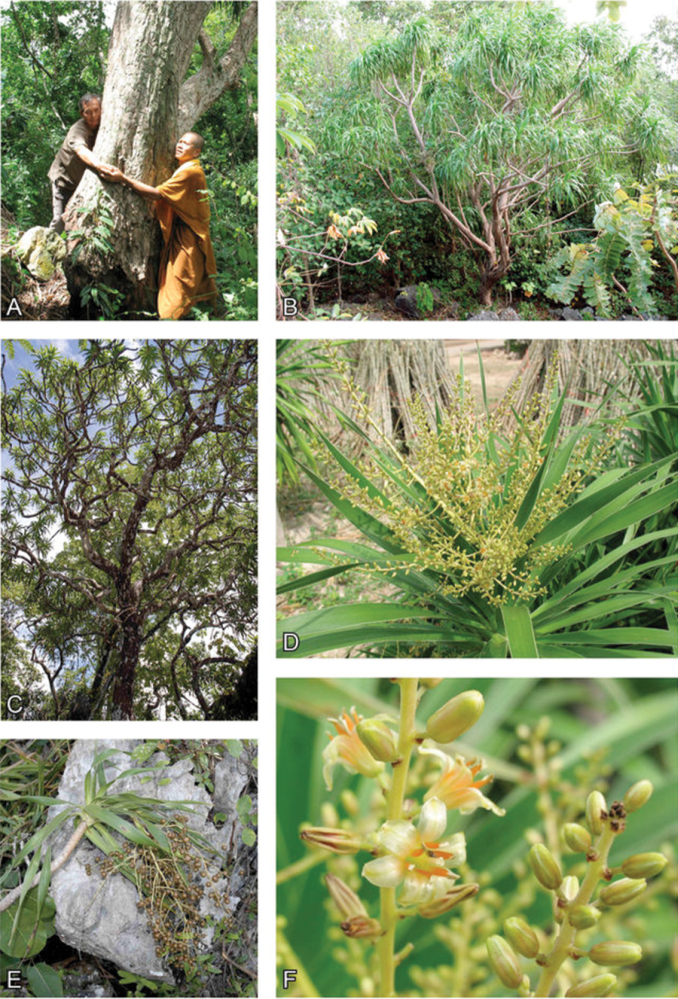
Photographs of *Dracaena kaweesakii* showing its habit and reproductive morphology. **A** Trunk base of large individual showing and corky, fissured surface **B** habit of relatively small individual **C** habit viewed from below showing the rich branching **D** Fertile shoot apex showing the white leaf margins and bearing an inflorescence **E** Branch apex with infructescence rendered pendent by weight of fruit, narrow branch diameter and leaf scars **F** Flowers on partial inflorescence showing tepal, stamen and gynoecium orientation, shape and colour and axis indumentum. Photographs by Warakorn Kasempankul/Parinya Siriponamat (**A, B, D, F**) and Paul Wilkin (**C, E**).

Thailand; Central, Lop Buri, Lam San Ti, Khao Wong Chan Daeng, 15°03'45.2"N, 101°27'17.1"E, fr. 26 May 2009, *Wilkin*, *Suksathan*, *Phonsena*, *Triboun*, *Keeratikiat* & *Plataan* 1500 (holotype QBG!; isotypes BKF!; K!;). Figs 1–3.

#### Description.

*Underground organs* unknown. *Indumentum* absent except for inflorescence axes and pedicels with tuberculate-villous trichomes to 0.15 mm long, often dense, sometimes crisped; similar trichomes on margins of leaves and tepal bases and margins. *Habit* treelike, 3–6 m(–12) m in height at maturity with an approximately equivalent crown diameter; usually much-branched, branches spreading and dividing, with up to several hundred terminal branches in large mature trees, branches rarely as few as ca 10 (mainly in high altitude forms such as those on Doi Chiang Dao). *Trunk* base to 1 m in diameter, sometimes markedly thicker than where branching begins, usually at ca 30–100 cm above the soil surface, epidermis brown, grey or ash-white, corky and fissured except at branch apices, enclosing cream-coloured, densely fibrous parenchyma, less dense towards centre, denser and heavier than that of *Dracaena jayniana*. Leaf scars present in apical 30–50 cm of branches where diam. is 1.3–2.4 cm (to 4.2 cm some cultivated plants e.g. Wilkinet al. 1521 perhaps due to increased nutrient availability), epidermis grey to light brown with tightly packed scars. *Leaves* in dense clusters of ca 20–50 leaves at shoot apices, clusters ca 30–120 cm in diam., 7–10 youngest leaves ascending to spreading, older recurved-pendent; divided into a basal sheath and blade, not pseudopetiolate; sheaths 8–16 × 17–32 mm, ovate to ovate-triangular, white (pale brown to mid brown when dry) sometimes with some irregular red markings from dried sap, sheath base clasping stem apex for ca 180°; leaf blades 11–60.5 × 0.9–3.1 cm, lorate-acuminate to linear-acuminate, not or weakly thickened above sheath, softer in texture than other Thai *Dracaena* species occurring on limestone, dark to mid green, thickly chartaceous to thinly coriaceous with a weak longitudinal central costa that is barely visible in apical half of the blade, primary venation parallel, dense sometimes denser in costa than elsewhere, secondary venation not visible; margins ca 1 mm broad and white when fresh, 0.1–0.3 mm wide when dry, to 0.5 mm on sheath, translucent white or pale brown, entire to bearing scattered trichomes, sometimes quite dense on sheath, often concealed by rolling of margins during drying; apex acuminate, terminated by a ca 1.5 mm long blunt, angular apiculus, translucent white to pale brown when dry, similar in appearance to margins. *Inflorescence* terminal on shoot, apical, erect or ascending relative to shoot growth direction in flower but can become pendent in fruit due to their mass, with 4 levels of branching; peduncle (0.8–)3.2–15.5 cm long, primary fertile axis ca 25–38 cm long; partial inflorescences racemose, with a primary axis and flowers in glomerular clusters or solitary towards axis apex; peduncular bracts to 8.7 cm long, foliaceous but with a reduced sheath, lower primary axis bracts to 7.5 cm long, foliaceous, becoming smaller towards apex, ovate-acuminate to broadly so and increasingly brown and scarious towards axis apex, morphologically continuous with bracts of secondary and tertiary axes and glomerular bracts, secondary branches 3.4–19.1 cm long, tertiary branches 1.9–6.2 cm long; glomerules (fourth level of inflorescence branching) composed of 1–3 flowers, internodes between glomerules to 10 mm in length, glomerular bracts 1.2–1.6 × 0.6–1.5 mm, ovate to broadly so, acuminate to acute, pale brown, scarious, clasping glomerule base, floral bracts like glomerular bracts but smaller and tending to be acute rather than acuminate, clasping pedicel base. *Flowers* patent to axis to ascending on a 1.2–2.0 × 0.3–0.7 mm, terete-angular pedicel, expanded and articulated at its apex; floral stalk absent, with 0.5–0.8 × 1.0–1.4 mm receptacle inserted directly on pedicel apex; tepals 6.0–8.5 × 1.2–2.6 mm long, narrowly oblong to oblong-elliptic or oblong-lanceolate, free almost to base, erect, with apical half recurved, cream with a green or yellow hue, paler and more translucent towards margins, greener towards apex and along thickened longitudinal costa, apex cucullate, acute to rounded, margins towards apex bearing a fringe of translucent trichomes; filaments 2.2–3.8 × 0.4–0.7 mm (free part), erect, narrowly lanceoloid, thickened, intense orange, green at base and where fused to tepal base, orange colour derived from bundles of heavily pigmented cells in translucent matrix; anthers dorsifixed, before anthesis 1.7–2.2 × 0.4–0.7(–1.0) mm, pale yellow, oblongoid; ovary 2.4–3.3 × 1.3–2.0 mm, ellipsoid to narrowly obovoid, pale green, 3-locular, with an apical swelling at the apex of each vertical loculicidal dehiscence line, swelling minutely verrucate and surrounding point of style insertion, style 2.5–3.3 mm long, erect, terete, white, stigma 0.5–0.8 mm in diam, 3-lobed, capitate. *Infructescence* usually with most bracts fallen, trichomes substantially persistent. *Fruit* a berry, tepal remains persistent at base, each berry bearing 1–3 seeds, 6.6–8.3 × 7–8.5 mm and (sub)globose (where 1-seeded), 7.0–8.2 × 9.9–11.3 mm (2-seeded) or 8.3–8.8 × 11.2–12.0 mm (3-seeded) and lobed, light to mid brown, becoming orange at maturity but most orange fruit already fallen, when dry with a paler cap around point of style base insertion sometimes bearing stylar remains. *Seeds* ca 6–7 mm in diam., globose to broadly triquetrous, pale brown, smooth but microreticulate.

**Figure 4.**
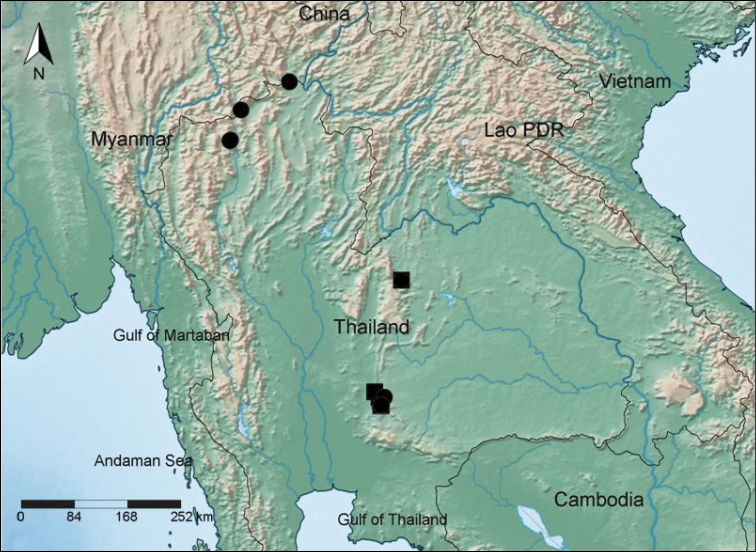
A map showing the distribution of *Dracaena kaweesakii* in northern, northeastern and central Thailand based on both specimen data (●) and observations (■). Map Created with SimpleMappr, http://www.simplemappr.net (Shorthouse 2010).

**Distribution**. Specimens seen from northern, northeastern and central Thailand, but ancedotal evidence exists as to extensive distribution in adjacent Burma (Fig. 4) through oral reports of the Burmese workers at Doi Ang Khang, which is on the Thailand/Burma border.

#### Specimens examined.

**Thailand.** Northern; Chiang Rai, Mae Sai, Doi Pha Mi/Doi Nang Non/Khun Nam Nang Non, 20°22'18.8"N, 99°51'12.9"E, old infructescence 1 June 2009, *Wilkin*, *Suksathan* & *Wongnak* 1507 (BKF!; K!; QBG!); Mae Sao, Doi Ang Khang Royal Project, cultivated bonsai garden nursery, 19°54'09.1"N, 99°02'30.2"E from a branch brought from a limestone ridge ca 2 km away (in Burma), sterile 30 May 2009, *Wilkin*, *Suksathan*, *Wongnak* & *Sumit* 1505 (BKF!; K!; QBG!); Chiang Mai, Doi Chiang Dao, sterile 28 Jan. 1913 *Kerr* 2870 (BM!; K!); Doi Chiang Dao, fr. 3 Nov. 1922, *Kerr* 6542 (BM! K!); Doi Chiang Dao (Chiengdao), fr. 22 April 1940, *Garrett* 1189 K!); Doi Chiang Dao, fr. 27 Sep. 1971, *Vidal* 5245 (AAU!; BKF!; L!; P!); Doi Chiang Dao, fr. 6 Jan. 1975, *Geesink, Hiepko & Phengklai* 8122 (K!, L!); Doi Chiang Dao Wildlife Sanctuary, north cliffs of Doi Nahng limestone mountain, fl. 17 March 1996, *Maxwell* 96-379 (BKF! CMU); Doi Chiang Dao, Doi Chiang Dao Wildlife Sanctuary, ridge to closest peak to ranger station, 19°23'02.3"N, 98°51'04.4"E, sterile 12 November 2011, *Wilkin*, *Suksathan*, *Trias Blasi*, *Clark* & *Phitak* 1525 (BKF!; K!; QBG!); Mae Sa, Queen Sirikit Botanic Garden Botanical Resort, near restaurant, cultivated plant of unknown origin, fr. 8 June 2009, *Wilkin, Suksathan & Sumit* 1521 (BKF!; K!; QBG!). Northeastern; Loei, Nong Hin, near Ban Suan Hom, 17°01'45.2"N, 101°44'28.8"E, fr. 5 June 2009, *Wilkin, Suksathan, Phonsena, Keeratikiat & Tot* 1517 (BKF!; K!; QBG!). Central; Saraburi, Muak Lek, Lam Phaya Klang, Si U-tumpon temple, fl. 23 Mar. 2009, *Sriponamat* 2 (BKF!); Saraburi, Muak Lek, Lam Phaya Klang, Si U-tumpon temple, fl. 7 May 2009, *Sriponamat* 3 (BKF!).

#### Vernacular names.

*Chan nuu* (Saraburi, Lop Buri, Loei) *Chan pa krai* (Chiang Mai, Chiang Rai), *Chan ku on* (Shan, Chiang Rai and Burma)

#### Ecology.

On limestone rocks from 550–2000 m altitude. In higher mountains on ridge tops (*Chan pa krai* form) and on the slopes and/or summits where they occur on lower limestone mountains in Loei and Lop Buri Provinces of Thailand. Higher altitude forms tend to be smaller, with fewer branches, a more open crown and smaller leaves, especially when in more exposed habitats. However, trees to ca 8 m in height and diam. and 50 cm DBH can be found in dense montane forest on limestone ridges at high altitude in northern Thailand.

#### Conservation status.

As indicated in Fig. 4, there are two groups of lowland populations of *Dracaena kaweesakii*, in Saraburi and Lop Buri (Central Thailand), where we have reports supported by photographs of seven populations in addition to the localities represented by *Wilkin* et al. 1500 and *Sriponamat* 2 and 3. It is found near Nong Hin in Loei Province (northeastern Thailand). There are three known localities in northern Thailand; at Doi Chiang Dao and near Doi Ang Khang and Mae Sai. These population locations were imported into GeoCAT (Bachman et al. 2011; http://geocat.kew.org/ ) and extent of occurrence (EOO) was calculated to be 73, 657 km^2^, while area of occupancy (AOO) was calculated to be 44 km^2^ based on a cell width of 2 km. However, anecdotal reports suggest the species is also distributed well into Burma on limestone ridges. The authors have seen populations of 10s to 100s of individuals and we have received reports of seven populations with sizes from eight to 150 mature plants in Saraburi and Lop Buri Provinces, suggesting that there are likely to be a few hundred plants in central Thailand. There appear to be few plants of Chan pa krai on Doi Chiang Dao. Thus it is likely that even including its distribution in Burma there would be less than 2, 500 mature individuals, the threshold for criterion C of EN (IUCN 2001).

*Dracaena kaweesakii* is extracted from the wild for use in horticulture in Thailand and is one of the more popular taxa due to its extensive branching. A number of populations are protected by proximity to temples or having been transplanted into their gardens. There is no evidence yet of over-extraction but sustainability studies are needed at population level; the authors have, for example, encountered an alley of vegetation being cleared to remove *Dracaena kaweesakii* from a limestone karst in Loei Province. Limestone habitats are generally threatened in Thailand by extraction for concrete manufacture, especially those closest to cities such as Bangkok; the populations in Saraburi and Lop Buri are the most vulnerable to this threat. Fires can also be problematic. Thus a preliminary assessment of Endangered (EN B2b (ii, iii, iv, v) C1) based on the criteria of IUCN (2001) is indicated.

#### Uses.

Used in horticulture in Thailand.

#### Etymology.

This species is named for our collaborator, friend and co-author Toi (Keeratkiat Kaweesak) to recognise of his extensive knowledge of *Chan* diversity.

#### Notes.

As indicated in Table 1, *Dracaena kaweesakii* differs from *Dracaena yuccifolia* in both vegetative and reproductive characters. It has up to several hundred branches, while the latter does not exceed about 80. The leaf sheaths are white (brown when dry) and lack the yellow or dark brown pigmentation found in *Dracaena kaweesakii*. The leaf blades of *Dracaena kaweesakii* possess a distinctive a narrow white margin when fresh. The inflorescence axis of *Dracaena kaweesakii* is tuberculate-villous not glabrous to microaculeate, and it lacks a floral stalk above the pedicel articulation; thus the flower is inserted directly at the pedicel apex. The tepals of *Dracaena kaweesakii* are cream-green or cream-yellow, with intense orange filaments (as opposed to bright white tepals and filaments), the anthers 1.7–2.2 mm long (as opposed to1.0–1.4 mm) and the style 2.2–3.3 mm long (as opposed to 3.4–5.5 mm). The ovary is broader (1.3–2.0 versus 0.5–1.1 mm) and fruits that largely remain brown on the infructescence, turning orange only just before or after falling (dull light red on the infructescence in *Dracaena yuccifolia*).

Two specimens collected on Hainan Island, *How* 70949 & *Lau* 225 may belong to an expanded concept of this species or a morphologically distinct close relative. Both have relatively thin leaves with pale margins and narrow terminal shoots; floral colour and dimensions also appear similar to those of *Dracaena kaweesakii* (e.g. tepals ca 6.5 mm long), although there are no flowers at anthesis on those specimens. They both differ in possessing glabrous inflorescence axes, up to 5 flowers per glomerule and a stalk above the point of articulation of the pedicel. Neither is *Dracaena cochinchinensis* as they have been determined. Both specimens possess a few leaves and an inflorescence with a few closed flowers. The taxonomy of *Dracaena* with free tepals in Hainan needs urgent revision including field-based study of fertile plants, not least to provide conservation status information.

The fruits of *Dracaena kaweesakii* were said to be dispersed by squirrels at Nong Hin in Loei Province. This may explain the late transition from brown to orange in colour around the time of fruit fall. Other species in Thailand have dull red fruits on the infructescence, bird dispersal appears likely. Field studies are needed to test these hypotheses.

## Supplementary Material

XML Treatment for Dracaena kaweesakii
